# Interleukin-4 in the Generation of the AERD Phenotype: Implications for Molecular Mechanisms Driving Therapeutic Benefit of Aspirin Desensitization

**DOI:** 10.1155/2012/182090

**Published:** 2012-01-03

**Authors:** John W. Steinke, Spencer C. Payne, Larry Borish

**Affiliations:** ^1^Asthma and Allergic Disease Center, Beirne Carter B. Center for Immunology Research, University of Virginia Health System, Charlottesville, VA 22908, USA; ^2^Department of Medicine, University of Virginia Health System, Charlottesville, VA 22908, USA; ^3^Department of Otolaryngology—Head and Neck Surgery, University of Virginia Health System, Charlottesville, VA 22908, USA

## Abstract

Aspirin-exacerbated respiratory disease (AERD) is explained in part by over-expression of 5-lipoxygenase, leukotriene C4 synthase (LTC_4_S) and the cysteinyl leukotriene (CysLT) receptors (CysLT1 and 2), resulting in constitutive over-production of CysLTs and the hyperresponsiveness to CysLTs that occurs with aspirin ingestion. Increased levels of IL-4 have been found in the sinus mucosa and nasal polyps of AERD subjects. Previous studies demonstrated that IL-4 is primarily responsible for the upregulation of LTC4S by mast cells and the upregulation of CysLT1 and 2 receptors on many immune cell types. Prostaglandin E_2_ (PGE_2_) acts to prevent CysLT secretion by inhibiting mast cell and eosinophil activation. PGE_2_ concentrations are reduced in AERD reflecting diminished expression of cyclooxygenase (COX)-2. IL-4 can inhibit basal and stimulated expression of COX-2 and microsomal PGE synthase 1 leading to decreased capacity for PGE_2_ secretion. Thus, IL-4 plays an important pathogenic role in generating the phenotype of AERD. This review will examine the evidence supporting this hypothesis and describe a model of how aspirin desensitization provides therapeutic benefit for AERD patients.

## 1. Introduction

Aspirin-exacerbated respiratory disease (AERD) is a syndrome characterized by asthma that, when present, is often severe and can be associated with aggressive airway remodeling [1], the presence of extensive hyperplastic eosinophilic sinusitis with nasal polyp (NP) formation [2], and intolerance to aspirin and other nonselective cyclooxygenase (COX) inhibitors [3–5]. A central feature of AERD is its association with profound overproduction and overresponsiveness to cysteinyl leukotrienes (CysLT) [6, 7]. These CysLTs have important proinflammatory and profibrotic effects that contribute both to the extensive hyperplastic sinusitis and nasal polyposis that characterize AERD and to the severity of these patients' asthma [1, 8, 9]. Various cytokines have been shown to modulate CysLT expression and responsiveness. This review will focus on the role of IL-4 in the induction and maintenance of the AERD phenotype and consider implications of aspirin desensitization in altering the leukotriene synthesis and responsiveness pathways.

## 2. Dysregulation of Cysteinyl Leukotriene Production in AERD

The overproduction of CysLTs in part reflects the increased expression of its primary synthesis enzymes 5-lipoxygenase (5-LO) and leukotriene C_4_ synthase (LTC_4_S). Upregulation of these enzymes is readily observed in the lungs and nasal polyps of AERD subjects [8, 10–12]. The overexpression of these enzymes results in constitutive excess production of the CysLTs as can be demonstrated in bronchoalveolar lavage samples or through quantification of urinary LTE_4_ [6, 7]. It is this upregulation of CysLT synthesis pathways that underlies the observed life-threatening surge in CysLT secretion following ingestion of aspirin or other non-steroidal anti-inflammatory drugs (NSAIDs) in AERD [13–16].

## 3. IL-4 Dysregulation of LTC_**4**_S

Several cell types including mononuclear phagocytes, basophils, mast cells, and eosinophils express LTC_4_S and are thereby capable of CysLT production and secretion. Mast cells typically express modest levels of LTC_4_S and its upregulation can be mediated by IL-4 (but not by IL-5 or IL-13) [17]. However, studies investigating the source of CysLTs in AERD have suggested that eosinophils might be the most important cell type driving the observed overexpression of LTC_4_S [11]. In our studies, we were not able to demonstrate a cytokine mechanism for increasing LTC_4_S expression in eosinophils. In part, this may reflect the short-lived nature of circulating eosinophils and their limited capacity for gene transcription and phenotypic modulation. Arguably, the “aspirin sensitive” phenotype of eosinophils in AERD reflects the impact of influences acting upon eosinophil progenitors in the bone marrow or, as increasingly recognized, long-lived transcriptionally active progenitors in the airway tissue itself [18].

## 4. IL-4 in AERD

Relatively little is known regarding the expression of IL-4 in sinus disease. The best study examined subjects with chronic hyperplastic eosinophilic sinusitis and nasal polyps, separating them on the basis of being allergic or nonallergic. IL-4 was prominently expressed in the tissue of the allergic subgroup when compared to either healthy controls or the nonallergic subgroup [19]. Examination of nasal secretions from subjects with chronic sinusitis found higher levels of IL-4 protein when compared with controls [20]. In another study that looked at allergic subjects with chronic sinusitis, IL-4 transcripts were found to be high in the ethmoid sinus mucosa and nasal turbinate tissue [21]. To our knowledge, however, specific expression of IL-4 in AERD has not been delineated. Our studies have demonstrated elevated levels of IL-4 expression at the mRNA and protein levels in AERD in comparison to control sinus tissue (unpublished results).

## 5. CysLT Receptor Dysregulation in AERD

AERD subjects also demonstrate markedly increased sensitivity to CysLTs [22], reflecting in part their upregulation of CysLT receptors [23]. The two well-characterized CysLT receptors can be distinguished by their relative potency for the CysLTs: CysLT1 receptor LTD_4_ > LTC_4_ ≫ LTE_4_ and CysLT2 receptor LTD_4_ = LTC_4_ ≫ LTE_4_. The relative insensitivity of either of these receptors to LTE_4_ is in contrast to the unique sensitivity of AERD subjects to this lipid mediator and has led to the suggestion that additional CysLT receptors must exist. This is more extensively reviewed elsewhere [24] and in the absence of definitive characterization, these will not be further addressed here. CysLT type 1 receptors are prominently expressed on airway smooth muscle [25] and these receptors primarily mediate the CysLT-induced bronchospasm associated with allergen exposure [26, 27]. The role of these receptors in bronchospasm following aspirin ingestion, however, is not clear. Our studies, and those of others, have shown varied distribution of the CysLT receptors on peripheral blood leukocytes [28–31]. While both receptors are widely expressed on eosinophils and mast cells only CysLT1 receptors can be found on neutrophils. Further, very few circulating T lymphocytes normally express either class of receptor (~4–8%) [28, 29, 31]. Interestingly, while the CysLT1 receptor has been found on lung fibroblasts as well, nasal polyp-derived fibroblasts express neither the CysLT1 nor 2 receptors [32].

## 6. IL-4 Dysregulation of CysLT1 and 2 Receptors

**Figure 1:**
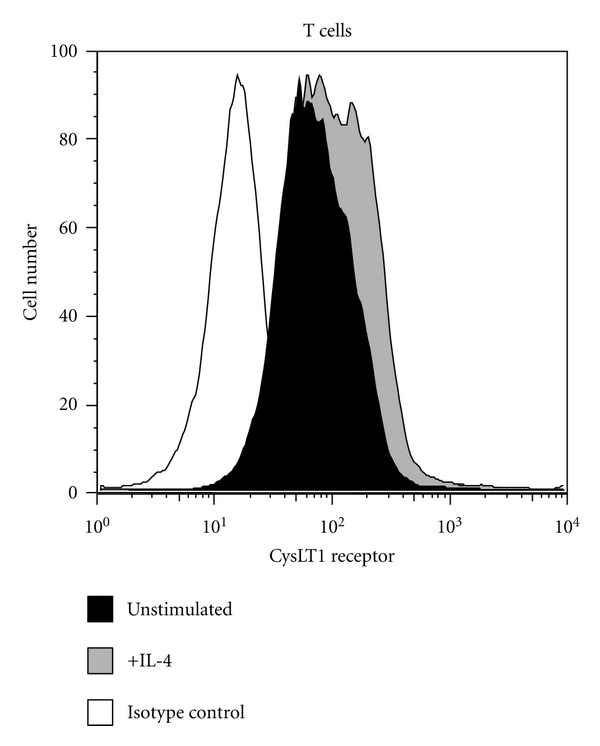
Cytokine modulation of cysteinyl leukotriene receptor protein expression on T cells. T cells were separated from blood using magnetic bead affinity chromatography and stimulated with 20 ng/mL IL-4 for 16 hrs before cells were collected for analysis. Cell surface expression of the CysLTR1 receptor was evaluated using rabbit polyclonal anti-CysLTR1 followed by labeling with FITC-conjugated goat anti-rabbit IgG. Isotype control is shown in white, unstimulated in black, and IL-4 stimulated in gray. Reprinted with permission of the American Thoracic Society [31].

As with LTC_4_S expression, the expression of the CysLT receptors is tightly regulated by cytokines, including, most prominently, IL-4. IL-4 upregulates cell surface expression of both CysLT1 and CysLT2 receptors on mast cells [33, 34]. Similarly, IL-4 stimulates cell surface expression of the CysLT1 receptor on monocytes and CysLT2 receptors on endothelial cells [35]. We investigated modulation by IL-4 of CysLT receptor expression on peripheral blood mononuclear cells and eosinophils [31]. The most impressive results were observed for IL-4 stimulation of the CysLT2 receptor. Significant increases in expression of CysLT2 receptor transcripts were seen on T and B lymphocytes, monocytes, and eosinophils. Additionally, IL-4 significantly upregulated CysLT1 receptor transcript and protein expression on T and B cells (Table 1 and Figure 1). This increased expression in secondary to IL-4 stimulation is mechanistically explained by the identification of a STAT6 response element in the CysLT1 receptor promoter region [36].

## 7. Prostaglandin (PG) E_**2**_ and PGE_**2**_ Receptor Dysregulation in AERD

In addition to modulation of CysLTs, the pathophysiology of AERD also involves downregulation of the prostaglandin synthesis pathway. PGE_2_ displays both pro- and anti-inflammatory functions reflecting its ability to interact with 4 distinct receptors (EP1–4) each having various activating or inhibitory functions. The ability of PGE_2_ acting through EP2 receptors to block eosinophil and mast cell degranulation is central to the pathogenesis of AERD and it has been shown that patients with AERD constitutively display low levels of PGE_2_ [12, 37]. The further reduction of tissue PGE_2_ concentrations by aspirin and other NSAIDs through COX inhibition precipitates the activation of these cells in AERD, and infusion of PGE_2_ protects against these non-IgE-mediated reactions [38, 39]. The sensitivity of AERD patients to low tissue PGE_2 _ concentrations is amplified by the reduced expression of the anti-inflammatory EP2 receptors also observed in this condition [40].

Several studies have investigated the mechanism behind the reduced levels of PGE2 in these patient and have indicated a correlation with a decrease in the responsible upstream enzymes. The production of PGE_2_ from arachidonic acid involves the sequential synthesis of PGG_2_/PGH_2_ by the two cyclooxygenase enzymes (COX-1 and COX-2) followed by the synthesis of PGE_2_ by the microsomal PGE_2_ synthases (mPGES-1, mPGES-2) and cytosolic PGE_2_ synthase (cPGES). COX-2 mRNA and protein expression are diminished in NPs of subjects with AERD [12, 41, 42]. Our studies have confirmed this diminished expression of COX-2 (Table 2 and [43]). We found no significant change in COX-1 or cPGES transcript expression but, along with COX-2, did show diminished expression of mPGES-2 and a trend towards diminished mPGES-1 expression (Table 2). It is mPGES-1 that is most relevant to PGE_2_ production in inflammatory disorders such as AERD as it is functionally coupled with COX-2 [44]. In general, mPGES-2 is thought to be primarily expressed by the heart and brain and the relevance of this dysregulation in AERD is unclear and may merely reflect the different histologies of control sinus epithelium and AERD NPs.

Diminished COX-2 expression and the reduced capacity to synthesize PGE_2_ contributes to the severity of inflammation observed in AERD and accentuates the sensitivity of these individuals to the inhibition of PGE_2_ synthesis associated with aspirin and other NSAIDs. This may also explain the paradoxical absence of symptoms in AERD patients that are typical in other forms of chronic sinusitis, such as pain and pressure. With this relative absence of COX-2, AERD subjects become dependent upon COX-1 for the PGE_2_ that is necessary to restrain mast cell and eosinophil activation. Most AERD patients tolerate selective COX-2 inhibitors supporting this concept regarding the unique importance of COX-1-derived PGE_2_.

## 8. IL-4 Dysregulation of PGE_**2**_ Synthesis Pathways

**Figure 2:**
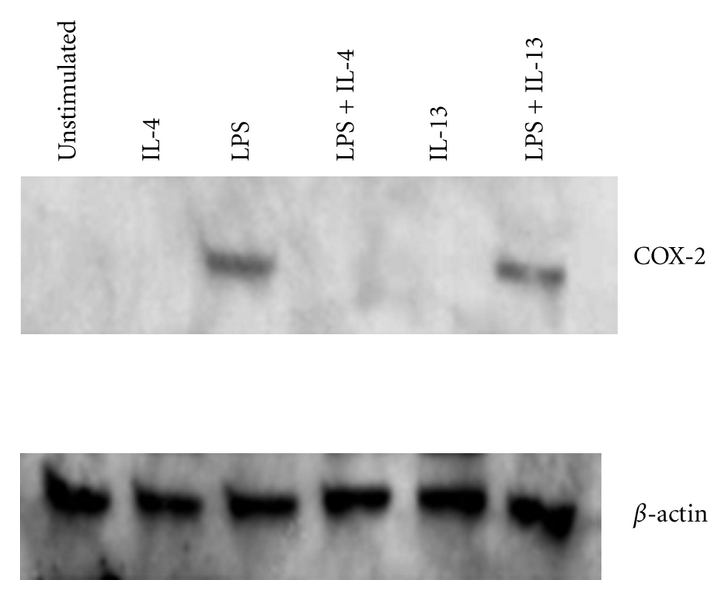
COX-2 protein expression in resting and LPS-stimulated monocytes. Monocytes were isolated from blood by magnetic bead purification. Cells were treated with IL-4 (10 ng/mL), IL-13 (10 ng/mL), or LPS (1 *μ*g/mL) for 24 hrs and whole cell lysates collected. Proteins were separated on a 10% SDS acrylamide gel and transferred to nitrocellulose. The membrane was probed with anti-COX-2 and then stripped and reprobed with anti-*β*-actin.

We investigated the molecular mechanism underlying inhibition of PGE_2_ synthesis pathways in AERD, focusing on influences of IL-4, reflecting again, its prominent expression in AERD, its previously described influences on the prostaglandin metabolic pathways [45, 46], and its involvement in the other facets of arachidonate dysregulation that have been previously discussed. For example, in contrast to IL-4 and despite being highly expressed in AERD, IL-5 did not influence PGE_2_ production or responsiveness in our studies (unpublished data). Our studies were performed on nasal polyp-derived fibroblasts, mononuclear phagocytic cells, and eosinophils. Monocytes were utilized both as representative inflammatory cells, but also because PGE_2_ is their dominant prostaglandin product. Significant inhibition of COX-2 and mPGES-1 (but not COX-1) mRNA expression was observed in response to IL-4 (Table 3). This appears to be a generalized effect of IL-4 insofar as similar inhibition was also observed in fibroblasts. Similar to IL-5, IL-13 also had no biological effect (not shown). No influence of IL-4 was observed in eosinophils. This may be a function of the low levels of COX-2 expressed in these cells, whose primary arachidonate product are the CysLTs, but again, may also reflect our use of terminally differentiated cells. The IL-4 effect on monocyte mRNA was further extended to expression of COX-2 protein as evaluated by Western blot. While low basal protein levels of COX-2 were observed, IL-4 (and not IL-13) inhibited LPS-stimulated COX-2 protein expression (Figure 2).

**Figure 3:**
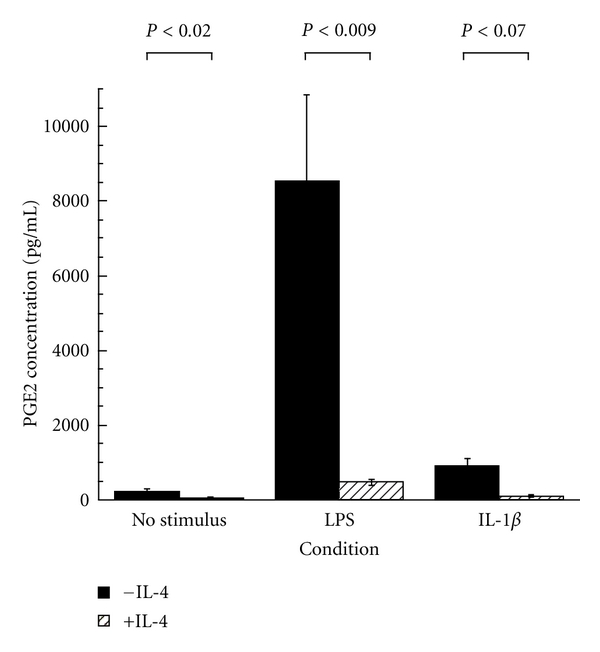
IL-4 inhibits monocyte PGE_2_ secretion. IL-4 was added to the cells (10 ng/mL) alone or either with LPS (1 *μ*g/mL) or IL-1*β* (10 ng/mL). Cells were incubated for 24 hrs before supernatants were collected. PGE_2_ levels were measured by ELISA and reported as pg/mL [43].

Inhibition of COX-2 and mPGES-1 synergize to result in dramatically less stimulated PGE_2_ secretion by monocytes (Figure 3). Thus, in addition to upregulating CysLT pathways, IL-4 contributes to the sensitivity of AERD patients by the inhibition of PGE_2_ production by aspirin/NSAIDs and, in particular, to nonselective (COX-1 and COX-2) inhibitors. However, it is necessary to remark that more than just loss of the tempering influences of PGE_2_ underlies these reactions, otherwise all asthmatics and, indeed, even healthy subjects would react to aspirin/NSAID ingestion with activation of their mast cells and eosinophils. Clearly, additional currently uncharacterized biochemical mechanisms, in addition to the relatively low levels of EP2 previously mentioned, must be uniquely driving the tendency of these compounds to trigger the mast cell, eosinophil, and perhaps other inflammatory cells in AERD.

## 9. Aspirin Desensitization for AERD—Implications for IL-4

Aspirin desensitization is an effective treatment for AERD and has been associated with diminished need for nasal endoscopic surgery, improved sense of smell, fewer bouts of acute sinusitis, reduced need for oral corticosteroids, and less severe asthma [3, 47, 48]. The molecular mechanism of the beneficial effects of aspirin has not been determined, but we believe this could be related to the ability of this compound to inhibit the biological activities of IL-4. Consistent with this concept are the observations that successful aspirin desensitization is associated with reversal of many of the IL-4-modulated features of AERD discussed above, including the ability of desensitization to downregulate both CysLT1 receptor expression [22, 23] and leukotriene synthesis [49] and reverse the inhibition of the PGE_2_ synthesis pathway; presumably by blocking the IL-4 induced-inhibition of mPGES-1 synthesis (Tanya Laidlaw, personal communication). The mechanism by which aspirin might block these effects is not immediately obvious. Aspirin (and other NSAIDs) are known to have off-target effects (effects not related to cyclooxygenase inhibition) including modulation of nuclear trafficking of numerous transcription factors such as NFAT, NF-*κ*B, and STAT6 [50–52]. Many of these off-target effects of aspirin only occur at significantly higher concentrations than those required for COX-1 or COX-2 inhibition [51]. The concentration of aspirin known to be effective after aspirin desensitization (up to 1300 mg/d) is somewhat higher than that required to inhibit COX-1 or COX-2, consistent with the concept that aspirin could be acting through one of these COX-independent pathways. Of relevance to our work, aspirin is known to directly inhibit T cell IL-4 expression [53]. Recently, continuous ingestion of aspirin for 6 months following desensitization has been shown to reduce sputum IL-4 levels [54]. Alternatively, we focused on recognition that engagement of the IL-4 receptor by IL-4 induces the activation of STAT6 via the Janus kinases. This STAT6 activation is critical for many of the biological activities of IL-4. As mentioned, a STAT6 site in the CysLT1 receptor promoter has been identified and was shown to be involved in IL-4-mediated transcription regulation [36]. Similarly, a putative STAT6 site has been identified in the LTC_4_S gene (Bing Lam, unpublished). Previous studies demonstrate that aspirin inhibits the activation of STAT6 [55]. These observations suggested to us that aspirin may produce its clinical utility in AERD through direct inhibition of the IL-4-activated STAT6 pathway. Aspirin hypersensitivity in AERD reflects inhibition of cyclooxygenase and as such also occurs with other COX inhibitors [56]. Although *desensitization* can be induced to other COX inhibitors, it is not established whether the therapeutic benefit that follows desensitization reflects COX inhibition or some of these other off-target anti-inflammatory effects of NSAIDs and salicylates.

## 10. Aspirin Modulation of STAT6 Nuclear Trafficking

**Figure 4:**
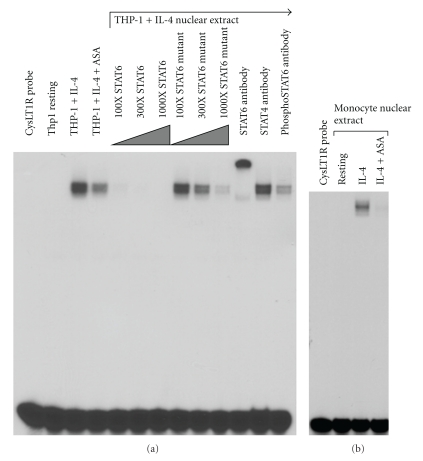
EMSA for STAT6. (a) EMSAs were performed using ^32^P-labeled oligomers comprising the STAT6 site within the CysLT1R promoter. Nuclear extracts were purified from THP-1 mononuclear cell lines in the resting state, IL-4 stimulated (10 ng/mL), and IL-4 stimulated in the additional presence of aspirin (10 mM). STAT6 binding was evaluated by performing EMSAs in the presence of 100–300-fold molar excess unlabeled STAT6 consensus sequence (comprising the *ε* heavy chain promoter) or a mutated STAT6 consensus sequence. EMSAs were also performed using STAT6, phosphoSTAT6, and, as a control, STAT4 antibodies. (b) Relevance to normal tissue was evaluated using nuclear extracts prepared as above, derived from enriched peripheral blood-derived mononuclear phagocytes [43].

Our studies investigated the inhibition by aspirin and other NSAIDs of the STAT6-mediated regulation of the CysLT1 receptor and LTC_4_S genes [43]. In a dose-dependent fashion, aspirin inhibited transcription of IL-4-induced CysLT1 receptor expression (not shown). Subsequently, via electrophoretic mobility shift assays (EMSA) we confirmed the presence of STAT6 binding-sites within both the CysLT1R (Figure 4) and LTC_4_S promoters (not shown).

**Figure 5:**
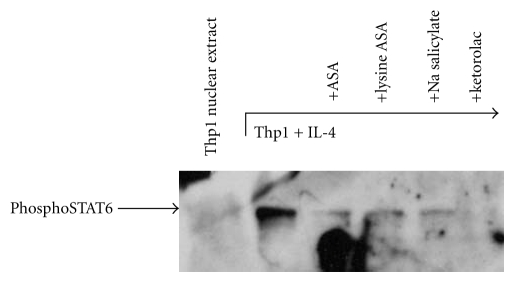
Western hybridization of nuclear extracts. Nuclear extracts obtained as described for Figure 4 were electrophoresed on a 10% SDS polyacrylamide gel and transferred to a nitrocellulose membrane. Presence of phosphoSTAT6 was determined via probing with anti-phosphoSTAT6 antibodies and a secondary peroxidase-labeled antibody [43].

 The presence of a mobility shift specifically mediated by STAT6 was confirmed by the ability of molar excess unlabeled DNA probes, comprising the STAT6 site within the *ε* heavy chain promoter, to block binding—but not a mutated version of this STAT6 probe. That this shift was produced by pSTAT6 was further confirmed using anti-STAT6 and anti-pSTAT6 antibodies. These results were extended to other NSAIDs including ketorolac, but not sodium salicylate (not shown). Ketorolac has been successfully utilized to diagnosis aspirin intolerance [56] and these observations suggest a plausible basis for it to have clinical efficacy in AERD. The mechanism by which aspirin blocks STAT6 expression is not known, but has been suggested to involve nuclear trafficking and recycling of transcription factors [51]. Our studies do not distinguish whether aspirin acts to either block induction of pSTAT6 or trafficking of newly activated pSTAT6 into the nucleus. However, our Western hybridization data do confirm the absence of functional pSTAT6 protein within the nuclei of aspirin-treated cells (Figure 5). Even more impressive, was the inhibition provided by ketorolac, supportive of recent findings regarding the efficacy of this compound in therapeutic desensitization of AERD [57]. These data thereby provide evidence that the CysLT1 receptor and LTC_4_S promoters have STAT6 binding sites that are occupied following IL-4 induction and inhibited by aspirin. As such, aspirin desensitization may provide effective therapy for AERD, at least in part, through mitigation of STAT6 activation, thereby downregulating the leukotriene pathways—as is observed clinically in successfully desensitized subjects.

 Countering the argument that aspirin may function in AERD as an IL-4-STAT6 antagonist is the lack of an obvious recognition of its utility in aspirin-tolerant asthma or even allergic rhinitis; disorders that arguably also involve IL-4 and STAT6 expression. It could be disputed that if aspirin is an effective anti-IL-4 agent it should be effective in all asthmatics. A subset of asthmatics is recognized who have aspirin-responsive asthma [58] and aspirin may have modest efficacy in patients with chronic sinusitis without aspirin intolerance (Donald Stevenson, unpublished). However, AERD is a distinct disorder from aspirin-tolerant asthma and these subjects are unique in their production of and sensitivity to leukotrienes. Efficacy of aspirin desensitization in AERD may therefore reflect the heightened importance of these leukotriene-dependent and, by extension, aspirin-responsive mechanisms in AERD. For example, leukotriene modifiers, in particular leukotriene synthesis inhibitors, seem uniquely efficacious in AERD in comparison to aspirin tolerant asthmatics [59]. Furthermore, the relative lack of efficacy of aspirin in aspirin-tolerant asthmatics, despite its putative ability to block IL-4, parallels the failure of IL-4-targeting biotherapeutics in these subjects (although suggests that perhaps these agents would have greater efficacy if used in AERD).

## 11. Summary

**Figure 6:**
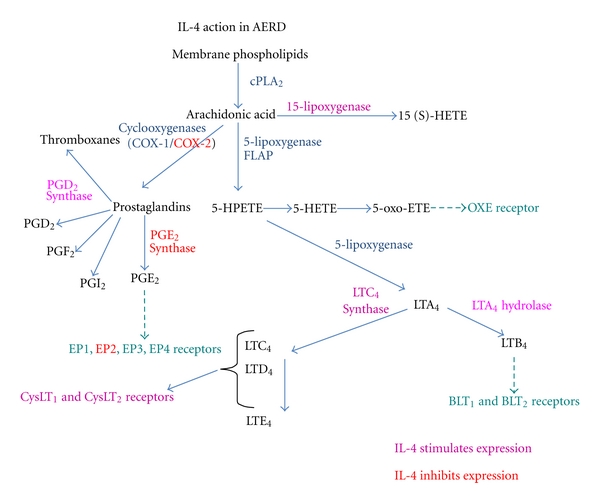
Summary of IL-4 activity on the leukotriene and prostaglandin synthesis pathways. Activation of gene synthesis by IL-4 is shown in pink while inhibition of gene synthesis by IL-4 is shown in red.

While the exact mechanisms driving AERD are not fully understood, part of the explanation is the marked overexpression of the 5-LO and LTC_4_S genes, resulting in constitutive overproduction of CysLTs, and the decrease in PGE_2_ production that prevents mast cell and eosinophil activation. These studies strongly suggest that AERD is derived, at least in part, from either the increased production or hyperresponsiveness to IL-4, although no obvious mechanism underlying this dysregulation has been identified. This increased expression of an IL-4 signature, as summarized in Figure 6, can have activating and inhibitory effects on gene expression in the LT and PG pathways. The net result of enhanced IL-4 levels is to increase synthesis of and responsiveness to LTs, while blocking production of protective PGE_2_. Compounds that target these molecules may lead to new therapeutic options for the treatment of AERD.

## Figures and Tables

**Table 1:** IL-4 modulation of CysLT receptor mRNA expression on leukocyte.

	CysLT1 receptor	CysLT2 receptor
Monocytes	1.6 ± 0.4	2.5 ± 0.7^†^
T lymphocytes	4.3 ± 2.1^†^	18.9 ± 10.1^§^
B lymphocytes	3.5 ± 0.6^†^	11.1 ± 2.7^†^
Eosinophils	2.4 ± 0.9	4.2 ± 1.5

*P < 0.05; ^†^ *P* < 0.01; ^§^ *P* < 0.001.

Quantitative polymerase chain reaction data, presented as fold change in comparison to unstimulated cells, which was set at 1. See legend for Table 2 for details.

**Table 2:** PgE_2_ metabolic pathway gene expression in control and AERD tissue.

Gene	Control ΔC_T_^1^	AERD ΔC_T_	ΔΔCT (2^ΔΔCT^)^2^
COX-1	10.5 ± 0.4^1^	11.3 ± 2.1	−0.8 (.57)
COX-2	6.0 ± 0.6	7.9 ± 2.8*	−1.9 (.27)
mPGES-1	4.0 ± 0.7	5.0 ± 0.5	−1 (.5)
mPGES-2	2.8 ± 0.8	4.5 ± 0.4*	−1.7 (.31)
cPGES	−0.1 ± 0.6	0.0 ± 0.5	+0.1 (1.07)

*P < 0.05.

^1^Quantitiatve polymerase chain reaction data are presented as ΔC_T_, which is the difference in threshold cycle of expression of each gene compared to housekeeping gene (each cycle corresponds to ~1 log_2_ difference in mRNA concentration; a higher ΔC_T_ represents less mRNA).

^2^ΔΔC_T_ is the difference in ΔC_T_ of gene expressed in control compared to AERD tissue.

2^ΔΔCT^ is the relative expression of gene in AERD compared to control tissue.

**Table 3:** IL-4 modulation of PgE_2_ metabolic pathway gene expression.

	COX-1	COX-2	mPGES-1
Monocytes	1.1 ± 0.7	0.3 ± 0.2^†^	0.2 ± 0.1^†^
Fibroblasts	0.6 ± 0.7	0.3 ± 0.1^†^	0.5 ± 0.1
Eosinophils	0.6 ± 0.6	0.7 ± 0.7	1.0 ± 0.5

Quantitative polymerase chain reaction data, presented as fold change in comparison to unstimulated cells, which was set at 1. ^†^ *P* < 0.01.

## Abbreviations

5-LO: 5-lipoxygenase
AERD: Aspirin-exacerbated respiratory disease
COX: Cyclooxygenase
CysLT: Cysteinyl leukotriene
LTC_4_S: Leukotriene C_4_ synthase
NF-*κ*B: Nuclear factor *κ*B
NFAT: Nuclear factor of activated T cells
NP: Nasal polyposis
NSAID: Nonsteroidal anti-inflammatory drugs
PG: Prostaglandin
PGES: Prostaglandin E_2_ synthase
STAT: Signal transducer and activator of transcription.
